# An embryo-specific expressing TGF-β family protein, growth-differentiation factor 3 (GDF3), augments progression of B16 melanoma

**DOI:** 10.1186/1756-9966-29-135

**Published:** 2010-10-15

**Authors:** Nobuyuki Ehira, Hiroyuki Oshiumi, Misako Matsumoto, Takeshi Kondo, Masahiro Asaka, Tsukasa Seya

**Affiliations:** 1 Department of Microbiology and Immunology, Graduate School of Medicine, Hokkaido University, Kita-15, Nishi-7, Kita-ku Sapporo 060-8638, Japan; 2 Department of Gastroenterology, Graduate School of Medicine, Hokkaido University, Kita-15, Nishi-7, Kita-ku Sapporo 060-8638, Japan

## Abstract

Malignant tumor cells often express embryonic antigens which share the expression with embryonic stem (ES) cells. The embryonic antigens are usually encoded by ES cell-specific genes, a number of which are associated with tumorigenesis and/or tumor progression. We examined the expression of ES cell-specific genes in the mouse B16 melanoma cell line to identify the factors promoting tumorigenesis. We found that endogenous growth-differentiation factor 3 (GDF3) expression was induced in implant B16 tumor during tumor progression in syngenic C57BL/6 mice. B16 F10, a subline with a high metastatic potential, continuously expressed GDF3 while low metastatic B16 F1 expressed comparatively decreased levels of GDF3. Overexpression of GDF3 promoted growth of implanted melanoma B16 F1 and F10 in syngenic mice. Ectopic expression of GDF3 was accompanied by an increased level of production of CD24/CD44. Such a profile was reported to be characteristic of melanoma stem cell-like cells. GDF3 expression was observed in embryonal carcinomas, primary testicular germ cell tumors, seminomas and breast carcinomas. However, the role of GDF3 in these cancers remains undetermined. Overexpression of GDF3 did not affect the growth of mouse hepatoma high or low metastatic sublines G5 or G1, both of which do not express GDF3. Since GDF3-driven CD24 acts as a receptor for endogenous innate immune ligands that modulate cell proliferation, CD24 is an effective determinant of tumorigenesis in malignant cell transformation. Finally, our results support the view that GDF3 has the ability to induce progression of CD24-inducible melanoma in mice.

## Introduction

Growth-differentiation factor 3 (GDF3) belongs to the transforming growth factor (TGF)-β superfamily, and is also called Vgr-2 [1,2]. Human GDF3 was first identified during a study of cDNAs expressed in human embryonal carcinoma cells [3]. GDF3 expression is also found in primary testicular germ cell tumors, seminomas, and breast carcinomas. Despite its ubiquitous expression the role of GDF3 in cancer remains undetermined [4-6]. In normal tissues, GDF3 is expressed in embryonic stem (ES) cells and the early embryo [7-10]. Chen et al. have demonstrated that mice with null mutation on GDF3 exhibit developmental abnormalities [11].

Cancers are composed of heterogeneous cell populations. The cancer stem cell (CSC) hypothesis was advocated for acute myeloid leukemia (AML) system [12] and recent studies have provided evidence that solid cancers can also originated from CSCs [13]. A previous report has shown that human melanomas also contain CSCs, and these tumor derived CSCs express ABCB5 [14]. This investigation also reported that the CSC population despite being very low could generate a tumor in human melanomas [14]. A recent work has shown that approximately 27% of human melanoma cells could initiate a tumor [15].

Mouse melanoma B16-F10 cells also contain CSC-like cells, which express CD133, CD44, and CD24 [16]. The mouse melanoma CSC-like cells, when injected subcutaneously into syngenic mice display tumorigenic ability [16]. Initial reports showed that the mouse CSC-like cells are a very small population, while most cells within the B16-F10 cell line retain the ability to induce malignancy [17].

The expression of ES-specific genes is observed in several human cancers. For example, the ES-specific gene, Sall4, is expressed in AML and precursor B-cell lymphoblastic leukemia [18,19]. Sall4 transgenic mice develop AML [19], but the molecular mechanism by which this occurs has not been shown yet. Another ES-specific gene, Klf4, functions as either a tumor suppressor or an oncogene in a tissue type or cell context dependent manner. Klf4 expression is frequently lost in colorectal [20], gastric [21], and bladder cancers [22]. Overexpression of Klf4 can reduce the tumorigenicity of colonic and gastric cancer cells *in vivo *[21,23]. On the other hand, high Klf4 expression levels have been detected in primary ductal carcinomas of the breast and oral squamous cell carcinomas [24,25], and ectopic expression of Klf4 induced squamous epithelial dysplasia in mice [26].

Because several ES-specific genes induce tumor progression, we tried to identify other ES-specific genes that promote tumorigenesis. Using mouse melanoma B16-F1 and B16-F10 cell lines as a model system, we found that GDF3 expression is different in these B16 sublines during tumor progression. We also observed that the ectopic expression of GDF3 promotes B16-F1 and B16-F10 tumorigensis. Interestingly, B16-F1 and B16-F10 cells induced expression of CD133, ABCB5, CD44 and CD24, which are expressed in mouse melanoma CSC-like cells during tumorigenesis, and ectopic generation of GDF3 increased the CD24 expression. Since CD24 is a pattern-recognition receptor to participate in poor prognosis in cancer patients, we discussed the possible role of the GDF3-CD24 pathway in tumor progression.

## Results

### The expression of ES cell-specific genes in mouse melanoma B16 cells

**Figure 1:**
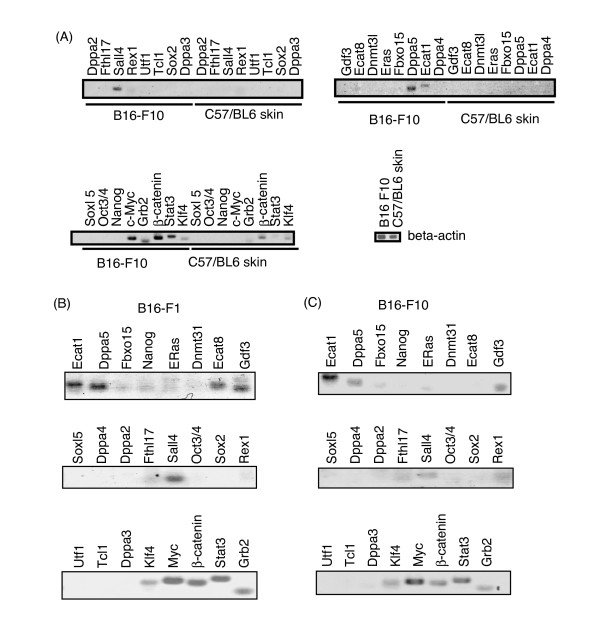
**Expression of ES-specific genes in mouse melanoma B16 cells**. (A) Total RNA was extracted from B16-F10 cells cultured in 10-cm dishes and RT-PCR was performed with primers listed in Table 1. Total RNA from excised C57BL/6 mice skin was used as control. B16-F10 cells expressed mRNA of Sall4, Dppa5, Ecat1, c-Myc, Grb2, β-catenin, and Stat3, which were not expressed in control C57/BL6 skin samples. (B, C) B16-F1 (B) or B16-F10 cells (C) were injected subcutaneously into C57BL/6 mice. Seven days after the injection, the tumor was excised. Total RNA was extracted and RT-PCR was performed. Two additional experiments resulted in similar profiles to that shown here.

We examined the expression of ES cell-specific genes in mouse melanoma B16 cell lines. The mouse melanoma B16-F10 cells were cultured in a 10-cm dish and their total RNA was extracted. Total RNA derived from excised C57BL/6 mouse skin was used as a control. RT-PCR analysis revealed that Sall4, Dppa5, Ecat1, and c-Myc were expressed in B16-F10 cells in culture dish but not in mouse skin (Figure 1A). In addition, Grb2, β-catenin, and Stat3 were expressed more in B16-F10 than in mouse skin (Figure 1A). Klf4 gene expression in B16-F10 cells was almost similar to that seen in mouse skin (Figure 1A). The expression of other genes was not detected under these experimental conditions (Figure 1A).

### Expression of ES-specific genes during tumorigenesis

Next, we examined the expression of ES-specific genes in B16 sublines during tumorigenesis. B16-F1 or B16-F10 cells were injected subcutaneously into C57BL/6 mice. Seven days after injection the tumor was excised and total RNA was extracted. RT-PCR analysis revealed that Ecat1, Dppa5, Ecat8, GDF3, Sall4, Klf4, c-Myc, β-catenin, Stat3, and Grb2 were expressed after tumorigenesis of B16-F1 and/or B16-F10 (Figure 1B,C).

**Figure 2:**
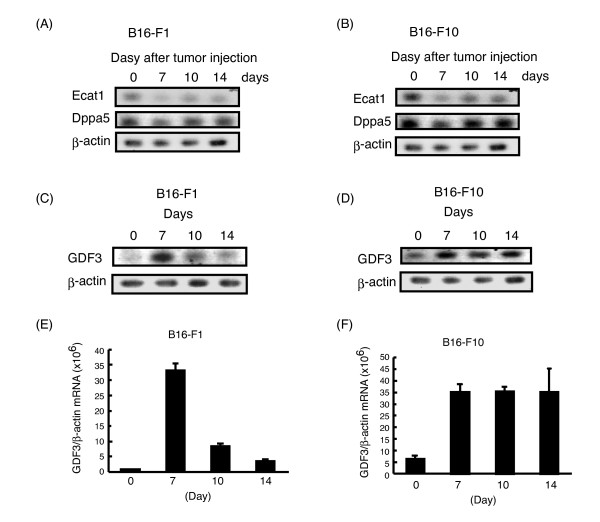
**Expression kinetics of Ecat1, Dppa5, and GDF3 during tumorigenesis**. B16-F1 and B16-F10 cells were injected subcutaneously into C57BL/6 mice. Tumors were excised on the indicated day. Total RNA was extracted from the tumor and RT-PCR (A-D) or RT-qPCR (E, F) was performed to detect Ecat1, Dppa5, and GDF3. (A, B) RT-PCR analyses revealed that mRNA of Eca1 and Dppa5 decreased during tumorigenesis. (C, E) In B16-F1 cells, GDF3 peaked at day 7 after tumor injection and then gradually decreased. (D, F). In contrast, GDF3 expression in B16-F10 cells increased 7 days after tumor injection and maintained a high level until 14 days after injection.

Sall4, Grb2, β-catenin, and Stat3 are known to be expressed in tumor cells and their roles in cancer has been already studied [19,27,28]. Ecat1, Dppa5, and GDF3 genes are expressed in ES cells, but their expression in tumor has not yet been reported. We initially focused on Ecat1 and Dppa5 during tumorigenesis. To investigate the expression kinetics we excised the B16-F1 or B16-F10 tumor 7, 10, or 14 days after implantation, and extracted total RNA. RT-PCR analysis revealed that Ecat1 and Dppa5 expression did not increase during tumorigenesis in both sublines (Figure 2A and 2B).

Next, we focused on GDF3. GDF3 mRNA expression was not detectable in B16-F1 cells cultured in dish (day 0 in Figure 2C) and only a weak expression was detected in B16-F10 cells cultured in dish (day 0 in Figure 2D). Interestingly, GDF3 mRNA expression increased approximately 10-fold 7 days after s.c. inoculation in both B16-F1 and B16-F10 cells (Figure 2C and 2D). Following the increase for 7 days after injection, GDF3 expression gradually decreased in B16F1 cells, but maintained a high level in B16-F10 cells (Figure 2E and 2F).

### GDF3 promotes the tumorigenesis of B16 melanoma

**Figure 3:**
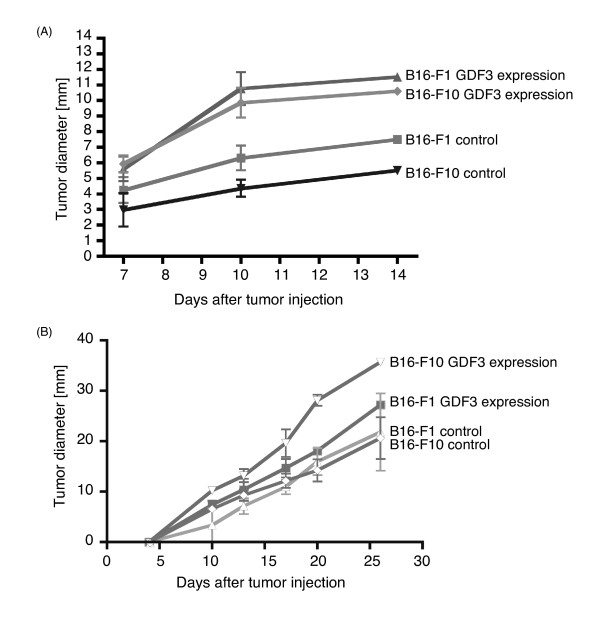
**Effect of GDF3 expression on B16 melanoma tumorigenesis**. B16-F1 and B16-F10 cells were transfected with empty or GDF3-expressing vectors. Twenty-four hours after transfection, 1 × 10^6 ^(A) or 1 × 10^5 ^(B) cells were injected subcutaneously into C57BL/6 mice and the tumor diameters were measured on the indicated day.

GDF3 is a member of TGF-β super family which is expressed in ES cells and in several human tumor cells. However, the role of GDF3 during tumorigenesis remains undetermined. To assess the functional importance of GDF3 expression during tumorigenesis, we examined whether the exogenous expression of GDF3 is sufficient to promote the tumorigenesis. Murine GDF3 cDNA was synthesized from the total RNA of B16-F1 cells and cloned into the pEF-BOS expression vector. The transfection efficiencies of this vector in B16- F1 and B16-F10 cells were ~25% with no difference between the two sublines. F1 or F10 cells were transfected with empty or GDF3-expressing vector. The following day, 1 × 10^6 ^of the transfected B16-F1 or B16-F10 cells were challenged subcutaneously into C57BL/6 mice and the tumor diameters were measured. The tumor diameters of the control B16-F1 tumors were larger than the control B16-F10 tumors at days 7, 10, and 14 (Figure 3A). Interestingly, the overexpression of GDF3 increased the tumor diameters in both B16-F1 and B16-F10 cells (Figure 3A). The promotion of tumorigenesis by GDF3 overexpression was also observed in mice injected with 1 × 10^5 ^of B16-F1 or B16-F10 cells (Figure 3B).

### GDF3 does not promote tumorigenesis of hepatoma G1 or G5 cells

**Figure 4:**
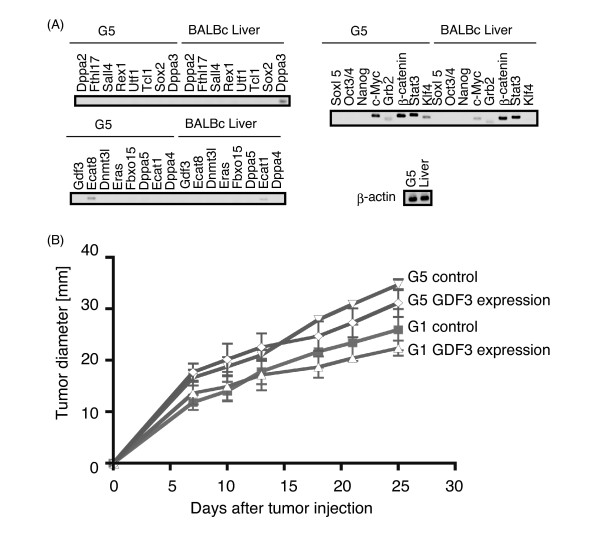
**Effect of GDF3 expression on mouse hepatoma G1 or G5 cells**. (A) Total RNA was extracted from G5 cells cultured in 10-cm dishes and BALB/c mouse liver, and RT-PCR analyses were carried out with primers listed in Table 1. (B) G1 and G5 cells were transfected with empty or GDF3-expressing vectors. Twenty-four hours after transfection, cells were injected subcutaneously into BALB/c mice and tumor diameters were measured on the indicated days. Two experiments with n = 4 were statistically analyzed.

The expression profiles of ES-specific genes from mouse hepatoma G5 cells were different from those from B16-F1 and B16-F10 cells (Figure 4A). We then examined the expression of GDF3 in mouse hepatoma G1 and G5 cell lines [29]. Unlike the mouse melanoma B16-F1 and B16-F10 cell lines, GDF3 expression was not observed in G1 or G5 cells in culture dish or in the cells during tumorigenesis (Figure 4A and data not shown).

To examine whether GDF3 promotes tumorigenesis of not only GDF3-expressing B16 melanomas but also tumors with no expression of GDF3, we transfected the mouse hepatoma G1 or G5 cell lines with empty or GDF3-expressing vectors, and injected the transfected cells into inbred BALB/c mice. Control transfected G1 or G5 cells formed tumors and the tumor size increased for 25 days (Figure 4B). Unlike B16 melanoma cells, forced expression of GDF3 did not result in acceleration of tumor growth in G1 or G5 cells (Figure 4B), indicating that the ability of GDF3 to promote tumorigenesis is specific to B16 melanoma that expresses GDF3 during s.c. progression.

### Expression of genes encoding melanoma CSCs markers

**Figure 5:**
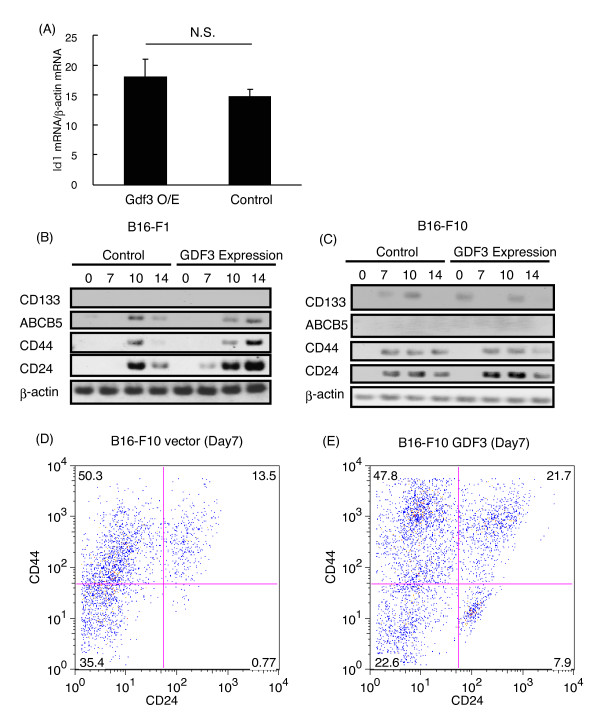
**(A) B16-F1 cells transfected with an empty vector or a GDF3-expressing vector**. Twenty-four hours after the transfection total RNA was extracted and RT-qPCR was performed to measure the Id1 expression. "N.S." stands for not statistically significant. (B, C) B16-F1 (B) or B16-F10 (C) cells were transfected with empty or GDF3-expressing vectors. Twenty-four hours after transfection cells were injected subcutaneously into C57BL/6 mice. Tumors were excised 7, 10, and 14 days after injection. Total RNA was extracted from tumors or cell from culture (day 0) and RT-PCR was performed. (D, E) B16-F10 cells were transfected with empty (D) or GDF3-expressing vectors (E) and 24 hours after the transfection cells were injected subcutaneously into C57BL/6 mice. The B16-F10 tumor was excised 7 days after injection. Cells were stained with a FITC-conjugated anti-CD24 antibody and a PE-conjugated anti-CD44 antibody. Cells were analyzed by FACS. One of three similar experiments is shown.

We examined the mechanism by which GDF3 accelerates tumor growth. GDF3 inhibits bone morphogenetic protein (BMP) signaling. Id1 is one of the transcription factors regulated by BMP signaling and its abnormal expression is observed in human cancers [27,30,31]. Therefore, we examined whether the GDF3 expression alters the Id1 expression; but no changes in Id1 expression was observed (Figure 5A).

ABCB5 is a marker of human melanoma CSCs, and CSCs with ABCB5 have a strong ability to generate tumors in xenotransplantation assays. Previously, Ning Gu and his colleagues showed that CD133-, CD44-, and CD24-positive B16-F10 cells show CSC-like feature and have strong ability to generate tumors [16]. We examined the expression of CD133, CD44, CD24, and ABCB5 during tumorigenesis of B16 melanoma cells transfected with empty or GDF3-expressing vectors. In B16-F1 cells, expression of ABCB5, CD44, and CD24 increased during tumorigenesis but CD133 expression was not observed at any time points (Figure 5B). Similar to B16-F1 cells, CD24 and CD44 expression increased during B16-F10 tumorigenesis but ABCB5 expression was not observed (Figure 5C). In contrast, CD133 expression was observed during B16-F10 tumorigenesis (Figure 5C). Production of GDF3 did not affect CD133, ABCB5, and CD44 expression. However, CD24 expression was higher in GDF3-transfected B16-F1 and B16-F10 cells compared to that of empty vector-transfected B16-F1 and B16-F10 cells (Figure 5B and 5C). These data indicate that GDF3 expression leads to increased CD24 mRNA expression or an increase in the fraction of cells expressing CD24 mRNA.

Next, we performed FACS analysis to detect CD24- and CD44-positive cells. B16-F10 cells transfected with empty or GDF3-expressing vector were injected subcutaneously into C57BL/6 mice. Seven days after injection, the tumor was excised, and the tumor cells were stained with anti-CD24 and -CD44 antibodies. FACS analysis showed that tumor cells with GDF3-expressing vector contained more CD24 and CD44 double-positive cells than those transfected with the empty vector (Figure 5D and 5E). These data indicate that the expression of GDF3 increase the number of CD24 and CD44 double-positive cells during tumorigenesis.

### **Expression levels of GDF3 in implant tumor cells**

**Figure 6:**
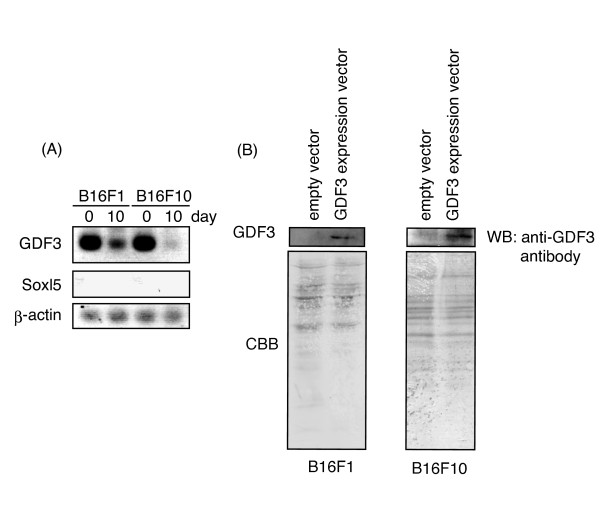
**(A) RT-PCR analysis of the GDF3 message in F1/F10 cells**. F1/F10 cells were transfected with the plasmid for expression of GDF3 (upper panel). Cells just before inoculation (indicated as 0 day) and cells isolated from tumors on day 10 after inoculation (indicated as day 10) were prepared and adjust the cell numbers. These cells were lysed and total RNA was extracted from the lysates. RT-PCR was performed to detect GDF3 as well as Soxl5 (nagative control, center panel) and β-actin (positive control, lower panel). PCR cycles are 32 rounds, 3 times less in those shown in Fig. 2C,D (B) Cell lysate (day 0) was subjected to SDS-PAGE (left 10% gel, right 8% gel) followed by immunoblotting. Lower panel- Commassie brilliant blue (CBB) staining of the blot. Upper panel- blots GDF3 band visualized by treating with anti-GDF3 mAb and then HRP-labeled 2^nd ^Ab. No relevant band of GDF3 was detected by CBB staining.

We finally confirmed that GDF3-transfected F1 and F10 cells continued to express GDF3 in implant tumors. RT-PCR analyses of excised tumors suggested that the transfected F1/F10 cells expressed the mRNA of GDF3 10 days after implantation although the levels of GDF3 mRNA decreased after 10 days compared to day 0 (Figure 6A). A negative control Soxl5 and a positive control β-actin were not affected by GDF3 transfection. Protein expression of GDF3 in F1 and F10 cells was examined by Western blotting using antibody against GDF3. A representative blotting profile is shown in Figure 6B. The protein as well as mRNA amounts of GDF3 were similar in F1 and F10 cells (Figure 6A,B). The results infer that the GDF3 message is translated into functional protein in these tumor cells and forced expression of GDF3 are still minimally expressed 10 days after transfection in these cells.

## Discussion

We have shown that GDF3 mRNA increased during tumorigenesis in mouse melanoma B16-F1 and B16-F10 cells. Although the genotypic and phenotypic differences of these sublines of the same cell line origin was described earlier [32], genes responsible for their tumorigenic difference have not been fully elucidated. We found that GDF3 overexpression promotes tumorigenesis of mouse melanoma by B16-F1 and B16-F-10 cells but not hepatoma by G1 or G5 cells. Moreover, ectopic expression of GDF3 increased CD24 expression in both B16-F1 and B16-F10 cells. Human GDF3 is primarily expressed in embryonal carcinomas, testicular germ cell tumors, seminomas, and breast carcinomas. However, the role of GDF3 in tumorigenesis has not been shown yet. This is the first report that establishes a positive role of GDF3 in tumorigenesis.

Mouse melanoma CSC-like cells have a high degree of tumorigenicity and express CD133, CD44, and CD24 [16]. The expression of these three genes increased during B16-F10 tumorigenesis, and B16-F1 cells expressed CD44, CD24, and ABCB5 during tumorigenesis. We were unable to isolate the cells expressing CD44, CD24, and CD133 (or ABCB5) from B16 tumors injected into syngenic mice because of the low percentage of these cells in the overall population. However, the expression of CD24, CD44 and CD133 (or ABCB5) in melanoma B16 cells implies that CSC-like cells emerge during tumorigenesis. Indeed, we observed more CD24 and CD44 double-positive cells in GDF3-expressing B16-F10 cells than in control B16-F10 cells during tumorigenesis. But we have not yet shown the mechanism by which GDF3 promotes turmorigenesis. The secondary effect of GDF3 expression on other genes should not be ruled out. One possible hypothesis is that GDF3 expression leads to an increase of some genes in CSC-like cells and these cells have a strong tumorigenic activity thus contributing to high GDF3 tumortigenicity.

Yamanaka and his colleagues firstly showed that the expression of four ES-specific genes, Klf4, Oct3/4, Sox2, and c-Myc, induces pluripotent stem cell proliferation from mouse embryonic and adult fibroblast cultures [10]. Another report also showed that another ES-specific gene Sall4 plays a positive role in the generation of pluripotent stem cells from blastocysts and fibroblasts [33]. In the current CSC theory, CSCs are derived from normal stem cells. Although several papers support this model, it is still unknown whether all CSCs are derived from normal stem cells [13]. In general, cancer cell genome becomes unstable because caretaker tumor suppressor genes are mutated during carcinogenesis [34]. Genome instability causes the expression of genes that are suppressed in normal tissues. In human ES cells, GDF3 supports the maintenance of the stem cell markers, Oct4, Nanog, and Sox2 [8,9]. Therefore, it is possible that some fraction of cancer cells may come to express these four genes *in vivo *leading to CSC formation from differentiated cancer cells, and GDF3 may promote this process. Another possibility of GDF3 role in tumorigensis is that GDF3 modulates TGF-mediated signaling, since it belongs to the TGF-β superfamily [8]. However, this model cannot explain why GDF3 expression increased only CD24 expression and not Id1 expression.

CD24 is a GPI-anchored sialoglycoprotein and is expressed in a variety of malignant cells [35]. CD24 participates in cell-cell contact and cell-matrix interaction and plays a role in cell proliferation. It is currently accepted that absence of CD24 on the tumor cell surface inhibits proliferative response and induces apoptosis in tumor cells, while up-regulation of CD24 promotes cell proliferation to increase tumor growth and metastasis [35,36]. Thus, the high CD24 level on tumor cells may predict poor prognosis in patients with cancer. In hepatocellular carcinoma CD24 level is actually correlated with patients' prognosis [36]. Our present finding furthers this notion and suggests that constitutive or forced expression of GDF3 in melanoma cells links the high CD24 expression accelerating tumor growth. By what mechanism TGF-β-like GDF3 induces up-regulation of CD24 on tumor cells, however, remains unknown.

In this regard, ectopic expression of GDF3 did not promote tumorigenesis of mouse hepatoma G1 and G5 cells. The expression profiles of CD24 in B16 melanoma sublines were parallel to those of GDF3, but hepatoma lines G1 and G5 had impaired the ability to induce GDF3-mediated CD24 expression. CD24 is rarely expressed on normal cells. Only limited subsets of myeloid cells are CD24-positive [36]. As the signal axis of this GDF3-derived CD24-inducing pathway is undetermined, it remains unsettled as to what is the molecular discrepancy between B16 F1/F10 melanoma cells and G-1/G5 hepatoma cells. Furthermore, the physiological role of the GDF3 signal and its downstream targets has not been elucidated. Yet the GDF3-CD24 pathway frequently turns positive when the cells are malignantly transformed [37] which may support the notion that CD24, when complexed with other molecules, alters its function for discrimination of danger signals [37].

Although possible experiments are in progress, another report suggests that CD24 is associated with Siglec-10 in humans or Siglec-G in mice serve as an innate immune receptor for endogenous self ligands named damage associated molecular pattern (DAMP) [38]. Accumulating evidence indicates that in tumor progression DAMP is released from damaged tissue or tumor cells and modulates both tumor and immune cells. Recent report suggested that the host inflammatory response to DAMP is partly controlled by a DAMP-CD24-Siglec axis [38]. We favor the speculation that the CD24 signals the presence of DAMP in a tumor micro environment, thereby augmenting inflammatory response to facilitate pathological tumor progression in GDF3-CD24 pathway-positive B16 F1/F10 but not -negative G-1/G-5 cells. Either way, this is the first report on the embryonic antigen GDF3 which is an inducer of CD24 and joins tumor cell proliferation. Further study may clarify the link between the CD24-Siglec G pathway and innate inflammatory response which occurs in invading tumor and facilitates to establish tumorigenesis.

## Materials and methods

### Cell lines and mice

B16-F1 and B16-F10 melanoma cells, G1 and G5 hepatoma cells were grown in RPMI1640 with 10% fetal bovine serum. These cell lines were transfectable, and transfection efficiencies were checked using the pEFBOS vector for expression of GFP. The transfection efficiencies were ~25% in F1 and F10 cells and ~20% in G-1 and G-5 cells (data not shown). We tried to establish stable clones constitutively expressing GDF3 in F1 and F10 cells, but failed to establish them. C57BL/6 and BALB/c mice (10-20 weeks of age) were purchased from Hokudo Co. (Sapporo, Japan). All mice were maintained under specific pathogen-free conditions in the animal facility of the Hokkaido University Graduate School of Medicine. Animal experiments were performed according to the guidelines set by the animal safety center, Japan.

### RT-PCR

Total RNA from cells, tumors and normal tissues was isolated using the TRIZOL reagent (Invitrogen) according to the manufacturer's standard instructions. Reverse transcription was performed with random primers using the High Capacity cDNA reverse transcription kit (ABI). PCR was performed using primers listed in Table 1. These primer sets are applicable to the detection of the messages in mouse ES cells [10]. PCR cycles were usually 35 rounds, and otherwise described. We avoided quantitative interpretation of the results of RT-PCR analysis. The amplified DNA fragments were analyzed with 1% agarose gel and stained with etidium bromide.

**Table 1:** Primer sequences

Primer name	Primer sequence (F: forward)	Primer sequence (R: reverse)
Dppa2	agaagccgtgcaaagaaaaa	gttaaaatgcaacgggctgt
Fthl17	actttgggactgtgggactg	ttgatagcatcctcgcactg
Sall4	gcccctcaactgtctctctg	gggagctgttttctccactg
Rex1	caggttctggaagcgagttc	gacaagcatgtgcttcctca
Utf1	ttacgagcaccgacactctg	cgaaggaacctcgtagatgc
Tcl1	caccatgagggacaagacct	cttacaccgctctgcaatca
Sox2	atgggctctgtggtcaagtc	ccctcccaattcccttgtat
Dppa3	ctttgttgtcggtgctgaaa	tcccgttcaaactcatttcc
Gdf3	acctttccaagatggctcct	cctgaaccacagacagagca
Ecat8	tgtgtactggcaaccaaaa	ctgaggtcccatcagctctc
Dnmt3l	caagcctcgtgactttcctc	ccatggcattgatcctctct
Eras	atcctaacccccaactgtcc	caagcctcgtgactttcctc
Fbxol5	ctatgattggctgcgacaga	gtagtgtcgggaggcaatgt
Dppa5	cagtcgctggtgctgaaata	tccatttagcccgaatcttg
Ecatl	gaatgcctggaagatccaaa	aaatctcagctcgcctttca
Dppa4	agggctttcccagaacaaat	gcaggtatctgctcctctgg
Soxl5	cggcgtaagagcaaaaactc	tgggatcactctgagggaag
Oct3/4	ccaatcagcttgggctagag	ctgggaaaggtgtccctgta
Nanog	cacccacccatgctagtctt	accctcaaactcctggtcct
c-Myc	gcccagtgaggatatcttgga	atcgcagatgaagctctggt
Grb2	tcaatgggaaagatggcttc	gagcatttcttctgccttgg
β-catenin	gtgcaattcctgagctgaca	cttaaagatggccagcaagc
Stat3	agactacaggccctcagcaa	cctctgtcaggaaaggcttg
CD133	ctcatgcttgagagatcaggc	cgttgaggaagatgtgcacc
CD24	actctcacttgaaattgggc	gcacatgttaattactagtaaagg
CD44	gaaaggcatcttatggatgtgc	ctgtagtgaaacacaacacc
ABCB5	gtggctgaagaagccttgtc	tgaagccgtagccctcttta
GDF3	aaatgtttgtgttgcggtca	tctggcacaggtgtcttcag

### Quantitative PCR

We used the following PCR primers: GDF3-F1, GDF3-R1, β-actin-F1, and β-actin R1 for quantitative PCR. Their sequences for GDF3 gene are listed in Table 1, and those of β-actin are a follows: β-actin-F1: TTT GCA GCT CCT TCG TTG C, and β-actin-R1: TCG TCA TCC ATG GCG AAC T. Quantitative PCR was performed by Step One real-time PCR system (ABI). The statistical comparisons were performed using the Student's *t *test between two groups.

### Tumor transplantation

B16 melanoma cells or G1, G5 hepatoma cells were cultured in 10-cm dishes and harvested with 0.02% EDTA solution. Cells were washed two times with D-PBS. Mice were anesthetized with diethyl ether and tumor cells were injected subcutaneously into C57BL/6 or BALB/c mice. Tumor volumes were measured using a caliper every 1 or 2 days. Tumor volume was calculated using the formula: Tumor volume (cm^3^) = (long diameter) × (short diameter) × (short diameter) × 0.4. Plotted data represent mean ± standard deviation (SD.).

### Flow cytometry

Flow cytometry (FACS) was performed using FACS caliber. Excised B16-F1 and B16-F10 tumors were treated with collagenase D for 30 minutes and then suspended in RPMI 1640 medium. Cells were washed two times with FACS buffer (1 × PBS, 1% BSA, 2 mM EDTA). 1 × 10^6 ^cells were suspended in 50 μl of FACS buffer. Anti mouse CD22 and CD 44 mouse antibody (eBioscience) were added into the cell suspension, and the cells were incubated at 4°C for 45 minutes. After the incubation cells were washed twice with PBS, and analyzed by FACS caliber.

### Western blot analysis

Cells were lysed in lysis buffer (20 mM Tris-HCl pH7.4, 150 mM NaCl, 1% NP-40, 10 mM EDTA, 25 mM iodoacetamide, 2 mM PMSF, protease inhibitor mixture (Roche)) and subjected to SDS-PAGE (8~10% gel) under reducing conditions followed by immunoblotting with anti-mouse GDF3 mAb or anti-β actin mAb (R&D Systems, Inc., Minneapolis, MN).

## Competing interests

The authors declare that they have no competing interests.

## Authors' contributions

HO, MM and TS designed the experiments. HO and NE carried out most of the experiments. TK and MA assigned this study to our laboratory. HO and TS wrote the manuscript. All authors read and approved the final manuscript.

## References

[B1] Jones CM Simon-Chazottes D Guenet JL Hogan BL Isolation of Vgr-2, a novel member of the transforming growth factor-beta-related gene family Mol Endocrinol 1992 61 1961 1968 10.1210/me.6.11.1961 1480182

[B2] McPherron AC Lee SJ GDF-3 and GDF-9: two new members of the transforming growth factor-beta superfamily containing a novel pattern of cysteines J Biol Chem 1993 268 3444 3449 8429021

[B3] Caricasole AA van Schaik RH Zeinstra LM Wierikx CD van Gurp RJ van den Pol M Looijenga LH Oosterhuis JW Pera MF Ward A de Bruijn D Kramer P de Jong FH van den Eijnden-van Raaij AJ Human growth-differentiation factor 3 (hGDF3): developmental regulation in human teratocarcinoma cell lines and expression in primary testicular germ cell tumours Oncogene 1998 16 95 103 10.1038/sj.onc.1201515 9467948

[B4] Ezeh UI Turek PJ Reijo RA Clark AT Human embryonic stem cell genes OCT4, NANOG, STELLAR, and GDF3 are expressed in both seminoma and breast carcinoma Cancer 2005 104 2255 2265 10.1002/cncr.21432 16228988

[B5] Skotheim RI Autio R Lind GE Kraggerud SM Andrews PW Monni O Kallioniemi O Lothe RA Novel genomic aberrations in testicular germ cell tumors by array-CGH, and associated gene expression changes Cell Oncol 2006 28 315 326 17167184 10.1155/2006/219786 PMC4615958

[B6] Gopalan A Dhall D Olgac S Fine SW Korkola JE Houldsworth J Chaganti RS Bosl GJ Reuter VE Tickoo SK Testicular mixed germ cell tumors: a morphological and immunohistochemical study using stem cell markers, OCT3/4, SOX2 and GDF3, with emphasis on morphologically difficult-to-classify areas Mod Pathol 2009 22 1066 1074 10.1038/modpathol.2009.66 19396148

[B7] Clark AT Rodriguez RT Bodnar MS Abeyta MJ Cedars MI Turek PJ Firpo MT Reijo Pera RA Human STELLAR, NANOG, and GDF3 genes are expressed in pluripotent cells and map to chromosome 12p13, a hotspot for teratocarcinoma Stem Cells 2004 22 169 179 10.1634/stemcells.22-2-169 14990856

[B8] Levine AJ Brivanlou AH GDF3 at the crossroads of TGF-beta signaling Cell Cycle 2006 5 1069 1073 16721050 10.4161/cc.5.10.2771

[B9] Levine AJ Brivanlou AH GDF3, a BMP inhibitor, regulates cell fate in stem cells and early embryos Development 2006 133 209 216 10.1242/dev.02192 16339188

[B10] Takahashi K Yamanaka S Induction of pluripotent stem cells from mouse embryonic and adult fibroblast cultures by defined factors Cell 2006 126 663 676 10.1016/j.cell.2006.07.024 16904174

[B11] Chen C Ware SM Sato A Houston-Hawkins DE Habas R Matzuk MM Shen MM Brown CW The Vg1-related protein Gdf3 acts in a Nodal signaling pathway in the pre-gastrulation mouse embryo Development 2006 133 319 329 10.1242/dev.02210 16368929

[B12] Lapidot T Sirard C Vormoor J Murdoch B Hoang T Caceres-Cortes J Minden M Paterson B Caligiuri MA Dick JE A cell initiating human acute myeloid leukaemia after transplantation into SCID mice Nature 1994 367 645 648 10.1038/367645a0 7509044

[B13] Visvader JE Lindeman GJ Cancer stem cells in solid tumours: accumulating evidence and unresolved questions Nat Rev Cancer 2008 8 755 768 10.1038/nrc2499 18784658

[B14] Schatton T Murphy GF Frank NY Yamaura K Waaga-Gasser AM Gasser M Zhan Q Jordan S Duncan LM Weishaupt C Fuhlbrigge RC Kupper TS Sayegh MH Frank MH Identification of cells initiating human melanomas Nature 2008 451 345 349 10.1038/nature06489 18202660 PMC3660705

[B15] Quintana E Shackleton M Sabel MS Fullen DR Johnson TM Morrison SJ Efficient tumour formation by single human melanoma cells Nature 2008 456 593 598 10.1038/nature07567 19052619 PMC2597380

[B16] Dou J Pan M Wen P Li Y Tang Q Chu L Zhao F Jiang C Hu W Hu K Gu N Isolation and identification of cancer stem-like cells from murine melanoma cell lines Cell Mol Immunol 2007 4 467 472 18163959

[B17] Zhong Y Guan K Zhou C Ma W Wang D Zhang Y Zhang S Cancer stem cells sustaining the growth of mouse melanoma are not rare Cancer Lett 2010 292 17 23 10.1016/j.canlet.2009.10.021 19944522

[B18] Cui W Kong NR Ma Y Amin HM Lai R Chai L Differential expression of the novel oncogene, SALL4, in lymphoma, plasma cell myeloma, and acute lymphoblastic leukemia Mod Pathol 2006 19 1585 1592 10.1038/modpathol.3800694 16998462

[B19] Ma Y Cui W Yang J Qu J Di C Amin HM Lai R Ritz J Krause DS Chai L SALL4, a novel oncogene, is constitutively expressed in human acute myeloid leukemia (AML) and induces AML in transgenic mice Blood 2006 108 2726 2735 10.1182/blood-2006-02-001594 16763212 PMC1895586

[B20] Zhao W Hisamuddin IM Nandan MO Babbin BA Lamb NE Yang VW Identification of Kruppel-like factor 4 as a potential tumor suppressor gene in colorectal cancer Oncogene 2004 23 395 402 10.1038/sj.onc.1207067 14724568 PMC1351029

[B21] Wei D Gong W Kanai M Schlunk C Wang L Yao JC Wu TT Huang S Xie K Drastic down-regulation of Kruppel-like factor 4 expression is critical in human gastric cancer development and progression Cancer Res 2005 65 2746 2754 10.1158/0008-5472.CAN-04-3619 15805274

[B22] Ohnishi S Ohnami S Laub F Aoki K Suzuki K Kanai Y Haga K Asaka M Ramirez F Yoshida T Downregulation and growth inhibitory effect of epithelial-type Kruppel-like transcription factor KLF4, but not KLF5, in bladder cancer Biochem Biophys Res Commun 2003 308 251 256 10.1016/S0006-291X(03)01356-1 12901861

[B23] Dang DT Chen X Feng J Torbenson M Dang LH Yang VW Overexpression of Kruppel-like factor 4 in the human colon cancer cell line RKO leads to reduced tumorigenecity Oncogene 2003 22 3424 3430 10.1038/sj.onc.1206413 12776194 PMC2275074

[B24] Pandya AY Talley LI Frost AR Fitzgerald TJ Trivedi V Chakravarthy M Chhieng DC Grizzle WE Engler JA Krontiras H Bland KI LoBuglio AF Lobo-Ruppert SM Ruppert JM Nuclear localization of KLF4 is associated with an aggressive phenotype in early-stage breast cancer Clin Cancer Res 2004 10 2709 2719 10.1158/1078-0432.CCR-03-0484 15102675

[B25] Chen YJ Wu CY Chang CC Ma CJ Li MC Chen CM Nuclear Kruppel-like factor 4 expression is associated with human skin squamous cell carcinoma progression and metastasis Cancer Biol Ther 2008 7 777 782 18376139 10.4161/cbt.7.5.5768

[B26] Foster KW Liu Z Nail CD Li X Fitzgerald TJ Bailey SK Frost AR Louro ID Townes TM Paterson AJ Kudlow JE Lobo-Ruppert SM Ruppert JM Induction of KLF4 in basal keratinocytes blocks the proliferation-differentiation switch and initiates squamous epithelial dysplasia Oncogene 2005 24 1491 1500 10.1038/sj.onc.1208307 15674344 PMC1361530

[B27] Ying QL Nichols J Chambers I Smith A BMP induction of Id proteins suppresses differentiation and sustains embryonic stem cell self-renewal in collaboration with STAT3 Cell 2003 115 281 292 10.1016/S0092-8674(03)00847-X 14636556

[B28] Giubellino A Burke TR Jr Bottaro DP. Grb2 signaling in cell motility and cancer Expert Opin Ther Targets 2008 12 1021 1033 10.1517/14728222.12.8.1021 18620523 PMC2764957

[B29] Saeki Y Seya T Hazeki K Ui M Hazeki O Akedo H Involvement of phosphoinositide 3-kinase in regulation of adhesive activity of highly metastatic hepatoma cells J Biochem 1998 124 1020 1025 9792928 10.1093/oxfordjournals.jbchem.a022194

[B30] Kang Y Chen CR Massague J A self-enabling TGFbeta response coupled to stress signaling: Smad engages stress response factor ATF3 for Id1 repression in epithelial cells Mol Cell 2003 11 915 926 10.1016/S1097-2765(03)00109-6 12718878

[B31] Schindl M Schoppmann SF Strobel T Heinzl H Leisser C Horvat R Birner P Level of Id-1 protein expression correlates with poor differentiation, enhanced malignant potential, and more aggressive clinical behavior of epithelial ovarian tumors Clin Cancer Res 2003 9 779 785 12576450

[B32] Ito A Watabe K Koma Y Kitamura Y An attempt to isolate genes responsible for spontaneous and experimental metastasis in the mouse model Histol Histopathol 2002 17 951 959 12168807 10.14670/HH-17.951

[B33] Tsubooka N Ichisaka T Okita K Takahashi K Nakagawa M Yamanaka S Roles of Sall4 in the generation of pluripotent stem cells from blastocysts and fibroblasts Genes Cells 2009 14 683 694 10.1111/j.1365-2443.2009.01301.x 19476507

[B34] Levitt NC Hickson ID Caretaker tumour suppressor genes that defend genome integrity Trends Mol Med 2002 8 179 186 10.1016/S1471-4914(02)02298-0 11927276

[B35] Kristiansen G Winzer KJ Mayordomo E Bellach J Schluns K Denkert C Dahl E Pilarsky C Altevogt P Guski H Dietel M CD24 expression is a new prognostic marker in breast cancer Clin Cancer Res 2003 9 4906 4913 14581365

[B36] Yang XR Xu Y Yu B Zhou J Li JC Qiu SJ Shi YH Wang XY Dai Z Shi GM Wu B Wu LM Yang GH Zhang BH Qin WX Fan J CD24 is a novel predictor for poor prognosis of hepatocellular carcinoma after surgery Clin Cancer Res 2009 15 5518 5527 10.1158/1078-0432.CCR-09-0151 19706825

[B37] Liu Y Chen GY Zheng P CD24-Siglec G/10 discriminates danger- from pathogen-associated molecular patterns Trends Immunol 2009 30 557 561 10.1016/j.it.2009.09.006 19786366 PMC2788100

[B38] Chen GY Tang J Zheng P Liu Y CD24 and Siglec-10 selectively repress tissue damage-induced immune responses Science 2009 323 1722 1725 10.1126/science.1168988 19264983 PMC2765686

