# Correction: an embryo-specific expressing TGF-β family protein, growth-differentiation factor 3 (GDF3), augments progression of B16 melanoma

**DOI:** 10.1186/1756-9966-33-22

**Published:** 2014-02-21

**Authors:** Nobuyuki Ehira, Hiroyuki Oshiumi, Misako Matsumoto, Takeshi Kondo, Masahiro Asaka, Tsukasa Seya

**Affiliations:** 1 Department of Microbiology and Immunology, Graduate School of Medicine, Hokkaido University, Kita-15, Nishi-7, Kita-ku, Sapporo 060-8638, Japan; 2 Department of Gastroenterology, Graduate School of Medicine, Hokkaido University, Kita-15, Nishi-7, Kita-ku, Sapporo 060-8638, Japan

## Correction

In Figure two of our article [1], panel A inadvertently contains a duplication of panel B. This error was made during the arrangement of the figure panels. The corrected, revised Figure two (Figure 1 here) is shown below. The error does not affect any conclusions drawn in the article. We regret any inconvenience this has caused.

**Figure 1:**
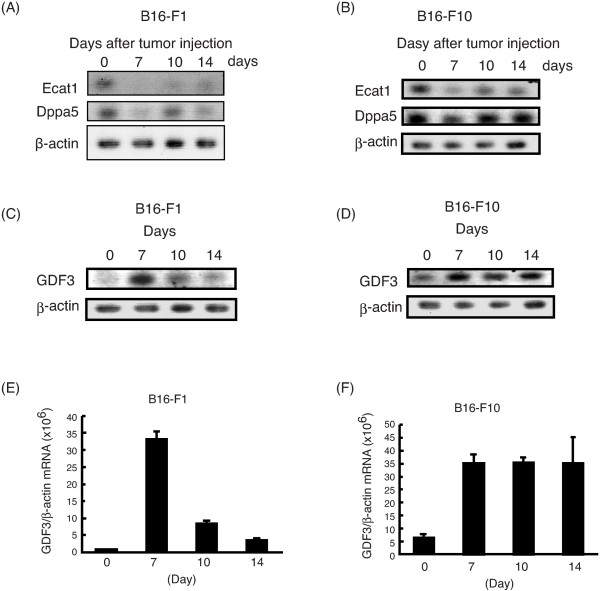
**Expression kinetics of Ecat1, Dppa5, and GDF3 during tumorigenesis.** B16-F1 and B16-F10 cells were injected subcutaneously into C57BL/6 mice. Tumors were excised on the indicated day. Total RNA was extracted from the tumor and RT-PCR **(A-D)** or RT-qPCR **(E, F)** was performed to detect Ecat1, Dppa5, and GDF3. **(A, B)** RT-PCR analyses revealed that mRNA of Eca1 and Dppa5 decreased during tumorigenesis. **(C, E)** In B16-F1 cells, GDF3 peaked at day 7 after tumor injection and then gradually decreased. **(D, F)**. In contrast, GDF3 expression in B16-F10 cells increased 7 days after tumor injection and maintained a high level until 14 days after injection.
